# Are autistic people disadvantaged by the criminal justice system? A case comparison

**DOI:** 10.1177/13623613221140284

**Published:** 2022-12-21

**Authors:** Rachel Slavny-Cross, Carrie Allison, Sarah Griffiths, Simon Baron-Cohen

**Affiliations:** 1 University of Cambridge, UK; 2 University College London (UCL), UK

**Keywords:** autism, criminal justice, meltdown, reasonable adjustments, shutdown

## Abstract

The UK government identified ‘improving support within the criminal justice systems’ as a priority for autistic people. There is a lack of quantitative research to enable policymakers to make evidence-based decisions on how to improve access to justice for autistic people. This study used a case-comparison design to compare the experiences of 145 autistic and 116 non-autistic adults who had been arrested at some point in their life. Autistic participants were nearly five times more likely to state that they were not given an appropriate adult even though they believed they needed one and felt less able to communicate with the police. Autistic participants felt less able to cope with stress, twice as likely to have experienced meltdowns and five times more likely to have experienced shutdowns because of their criminal justice involvement. We conclude that there are inequalities that autistic people face when navigating the criminal justice system and the degree to which they can participate effectively in the justice process. This has an impact on their mental health. Policy recommendations are discussed based on this evidence.

**Lay Abstract:**

Most autistic people will never experience being arrested or charged with a crime, however for those who do tend to be less satisfied with the way they were treated. The purpose of this study was to find out if autistic people are being disadvantaged by the criminal justice system if they are arrested. Previous research has shown that autistic people may have difficulties communicating with the police. This study builds on this knowledge by uncovering why autistic people may not feel able to communicate with the police and whether the police made any adjustments to help them. This study also measures the impact of being involved with the criminal justice system on autistic people’s mental health, such as stress, meltdowns and shutdowns. The results show that autistic people were not always given the support they felt they needed. For example, not all autistic people had an appropriate adult with them at the police station who could help to make sure they understood what was happening around them. Autistic people were also more likely to feel less able to cope with the stress and more likely to suffer meltdowns and shutdowns because of their involvement with the criminal justice system. We hope this study will help police officers and lawyers to better support autistic people if they become involved with the criminal justice system.

## Introduction

A study of young adults found few differences in rates of criminal justice system (CJS) involvement among young autistic adults, those with an intellectual disability and the general population (Yu et al., 2021). Young autistic adults were less likely to be involved in the CJS than their peers (Yu et al., 2021), and other studies indicate that autistic people are less likely to be offenders and more likely to be victims of crime (Beadle-Brown, Guest, Richardson, et al., 2014). However, one study reported that 19.5% of autistic youth had been stopped and questioned by police by the time they were in their early 20s (Rava et al., 2017). A previous study (Griffiths et al., 2019) reported that 18% of autistic adults had been stopped or arrested by the police. If an autistic person does become involved in the CJS, they may experience worse outcomes as a result of their social communication style and may be perceived unfavourably during proceedings, resulting in harsher penalties (Foster & Young, 2022). The UK government identified ‘improving support within the criminal and youth justice systems’ as a priority in their national strategy for autistic children, young people and adults (HM Government, 2021). However, there is a lack of quantitative research in this area to enable policymakers to make evidence-based decisions on how to improve access to justice for autistic people.

If autistic people do become involved with the CJS, they report unfavourable experiences and fears around future police involvement (Salerno & Schuller, 2019; Salerno-Ferraro & Schuller, 2020). Some autistic people and their carers worry that behaviours such as stimming or communication difficulties could be misinterpreted by the police and lead to adverse outcomes following police involvement (Salerno-Ferraro and Schuller (2020). Report concerns that autistic behaviours may be perceived as being non-compliant and that aggressive behaviours may emerge during police interactions, resulting in the misuse of police force (Wallace et al., 2021).

There are some aspects of the cognitive phenotype of autism which might increase the risk of being arrested for an alleged crime. These include obsessive interests, social naivete, difficulties with cognitive empathy or ‘theory of mind’, and using an if-and-then logic to problem-solve their way out of a dangerous situation when a non-autistic person would perhaps have taken advice from a friend or family member or advisor or lawyer, or other social sources of help (Baron-Cohen, 2020).

There are some qualitative studies exploring autistic people’s experiences in the CJS that indicate that the CJS is not serving all autistic people and it could. A recent report found four themes that emerged from interviews with police and autistic people including (1) the misinterpretation of autistic behaviours, (2) the need for an identification system and disclosure of autism diagnosis prior to encounters, (3) the need for autism training among police officers and (4) the importance of building community connections between the police and the autistic community (Railey et al., 2020). Other qualitative studies report similar experiences and recommendations (Gibbs & Haas, 2020; Haas & Gibbs, 2021). The current literature is based largely on qualitative data or from autistic people and their families, without a comparison group. There is currently no literature that aims to understand the difficulties autistic people face during CJS involvement in comparison with the experiences of non-autistic people going through the same process. The difficulties that may be unique to autistic people are poorly understood, and to date, there are no studies that aim to quantify the inequalities autistic people face relative to non-autistic individuals navigating the CJS.

The Police and Criminal Evidence Act 1984 in the United Kingdom states that any person suspected of a criminal offence below the age of 18 years or a vulnerable adult (i.e. has a disability or mental health condition) must be offered an appropriate adult (AA) during police questioning (Home Office, 1984). AAs act to safeguard the interests and rights of vulnerable detainees by ensuring that they are treated in a just manner and are able to participate effectively during an investigation. In the United States, there is no equivalent to ‘AAs’; however, the American Disability Act states that reasonable modifications to policies, practices and procedures should be made for individuals with disabilities (ADA, 1990). Reasonable adjustments are typically implemented with the aim of facilitating effective communication between vulnerable adults and professionals within the CJS. However, little is known about how frequently these adjustments are implemented, and how effective they are in terms of detainees feeling supported and able to communicate with professionals.

Navigating the CJS is often a stressful life event that can have considerable impact on a person’s mental health. Autistic people are particularly vulnerable to adverse mental health outcomes and are more likely to engage in self-harm and have a history of suicide attempts (Hirvikoski et al., 2020), with 66% reporting suicidal ideation (Cassidy et al., 2014). A study we conducted with criminal lawyers revealed concerns over the mental well-being of autistic adults involved with the CJS (Slavny-Cross et al., 2022). This was the first study to explore self-harm and suicidality during CJS involvement among autistic people. Further research is needed to gather data from autistic people themselves to understand the mental health risks they face when navigating such a uniquely stressful event.

This study aimed to address the gaps in the literature using a case-comparison design to compare the experiences of autistic and non-autistic people who have had involvement with the CJS. This was to identify any inequalities that may be unique to autistic people. We aimed to answer the following three questions:

1. Are autistic people as satisfied with their treatment by the CJS as non-autistic people?
2. Do autistic people access reasonable adjustments and the support they need during their involvement with the CJS?
3. Do autistic people experience similar impact on their mental health during their involvement with the CJS as non-autistic people?

## Method

### Participants

Participants were 145 autistic and 116 non-autistic adults (above 18 years of age) who had been arrested at some point in their lifetime. Adults with an autism diagnosis from a medical professional (*n* = 111) and those without a diagnosis but who self-identify as autistic (*n* = 34) were recruited to take part in this study. The 261 participants who took part in the survey resided in the United Kingdom and Northern Ireland (85%), the United States (8%), Ireland (4%), Canada (1%), Germany (0.5%), Hungary (0.5%), Australia (0.5%), Saudi Arabia (0.5%) and Sweden (0.5%). Participants from different geographical locations were pooled to improve the generalisability of the data. Table 1 shows offence categories of charges following arrest by group, which were broadly similar among the groups. Table 2 reports demographic information on the participants by group. Autistic participants (those with an autism diagnosis and those self-diagnosed were pooled) were more likely than non-autistic participants to have a diagnosis other than autism, χ^2^(1, *N* = 257) = 37.98, *p* > 0.001.

**Table 1.** Offence categories of charges made by group.

Offence category	Autism	Self-diagnosed autism	Non-autistic
Fraud, cybercrime, hacking	1% (1/111)	6% (2/34)	5% (6/116)
Harassment	3% (3/111)	6% (2/34)	1% (1/116)
Sexual offences	6% (7/111)	15% (5/34)	5% (6/116)
Less serious violent offences (e.g. common assault)	14% (15/111)	12% (4/34)	12% (14/116)
Serious violent offences (e.g. wounding with intent, murder)	3% (3/111)	3% (1/34)	3% (3/116)
Theft or robbery	9% (10/111)	12% (4/34)	8% (9/116)
Vandalism including arson	5% (5/111)	0% (0/34)	7% (8/116)
Drug offences	6% (7/111)	6% (2/34)	11% (13/116)
Possession of weapon	5% (5/111)	3% (1/34)	2% (2/116)
Dangerous driving	4% (4/111)	9% (3/34)	8% (9/116)
Other offences	9% (10/111)	9% (3/34)	7% (8/116)
Missing data	41% (45/111)	24% (8/34)	39% (45/116)

Note. Data are *n*/*N* (%), for which *n* is the number of participants charged with the offence and *N* is the total number of participants in the group. Some participants had charges that fell within more than one offence category (eight non-autistic participants, one self-diagnosed autistic participant and four autistic participants).

**Table 2.** Demographic information by group.

	Autism (*N* = 111)	Self-diagnosed autism (*N* = 34)	Non-autistic (*N* = 116)
Age at first arrest			
*M* (*SD*)	25.18 (12.82)^a^	25.15 (12.68)^b^	22.33 (9.37)
Age left full-time education			
*M* (*SD*)	19.75 (4.02)^c^	22.47 (5.10)	19.44 (3.86)^d^
Sex			
Male	66% (73/111)	65% (22/34)	76% (88/116)
Female	33% (37/111)	35% (12/34)	22% (25/116)
Missing data	1% (1/111)	0% (0/34)	2% (3/116)
Gender			
Female	29% (32/111)	32% (11/34)	22% (26/116)
Male	67% (74/111)	65% (22/34)	75% (87/116)
Non-binary	4% (4/111)	3% (1/34)	0% (0/116)
Other	1% (1/111)	0% (0/34)	3% (3/116)
Ethnicity			
White	86% (96/111)	88% (30/34)	89% (103/116)
Other	14% (15/111)	12% (4/34)	11% (13/116)
Diagnosis			
Intellectual disability	7% (8/111)	9% (3/34)	2% (2/116)
Mental health disability	63% (70/111)	56% (19/34)	47% (55/116)
Neurodevelopmental disability^e^	44% (49/111)	3% (1/34)	9% (10/116)
Physical disability	14% (16/111)	6% (2/34)	10% (12/116)
No diagnosis^e^	0% (0/111)	35% (12/34)	40% (46/116)
Missing data	0% (0/111)	3% (1/34)	3% (3/116)

Data are *n*/*N* (%); Some participants fell within more than one diagnosis category (12 in the non-autistic group, 4 in the self-diagnosed autism group and 32 in the autism group).

*SD*: standard deviation.

a *N* = 106 due to missing data from five participants.

b *N* = 33 due to missing data from one participant.

c *N* = 107 due to missing data from four participants.

d *N* = 115 due to missing data from one participant.

e Other than an autism spectrum condition for participants in the autism group.

Participants were recruited via adverts placed on social media (e.g. Twitter, Facebook and Reddit) and through the Cambridge Autism Research Database (CARD) (www.autismresearchcentre.com). Written informed consent was obtained in an online tick box format from all participants. All procedures contributing to this work comply with the ethical standards of the relevant national and institutional committees on human experimentation and with the Helsinki Declaration of 1975, as revised in 2008. This study was approved by the local university research ethics committee.

### Measures

Data were collected via an online survey using Qualtrics software, version (May 2021), copyright^©^ (2021) Qualtrics. The survey was part of a broader piece of work on autism and the CJS. A related article from the perspective of lawyers has already been published (Slavny-Cross et al., 2022). This article focuses on the experiences of autistic people with respect to reasonable adjustments, support and mental well-being during CJS involvement, and items from the survey measuring risk factors for CJS involvement are not included in the current study. The survey was developed through consultation with autistic people who had experience with the CJS to ensure the focus and content was relevant to autistic people’s experiences. The survey consisted of multiple-choice questions covering (1) demographic information (11 items), (2) lifetime experience of the CJS (8 items), (3) alleged crime/s (14 items), (4) experience at the police station (9 items), (5) their trial(s) if they had had one (4 items) and finally, (6) their mental well-being (7 items). Some items were only visible to participants if they answered ‘yes’ to certain items. To address the differences in legislation and policies across the various demographic locations in which our participants resided, we included survey questions that were broad enough to cover common legal frameworks. For example, we included the following definition of an AA in the survey to account for any independent person who provided support during police interviews: ‘An appropriate adult can be a carer or relative, or sometimes a trained professional who you have never met before. Appropriate adults are there to support you at the police station and make sure you understand your rights’. A ‘Don’t know’ answer option was also included for all items to account for questions that were not applicable to participants. The survey took most participants between 10 and 20 min to complete.

For items that had a ‘yes’, ‘no’ or ‘don’t know’ option, data were scored as 1 for ‘yes’, 0 for ‘no’ and ‘don’t know’ was scored as missing data. For Likert-type scale items (e.g. ‘How satisfied were you that you were treated fairly by police?’), data were scored as 1 for *extremely dissatisfied*, 2 for *somewhat dissatisfied*, 3 for *neither satisfied nor dissatisfied*, 4 for *somewhat satisfied* and 5 for *extremely satisfied*.

### Design and analysis

Autistic and non-autistic groups were compared using a case-comparison design. Data were first analysed excluding those who self-diagnosed as autistic and then subsequently analysed using a merged autism group including both the self-diagnosed and the diagnosed group. None of the findings differed significantly when including the self-diagnosed participants in the autism group; therefore, the results reported are for the pooled autism group. Bonferroni corrections were applied to *p*-values to correct for multiple comparisons across analyses related to individual research questions, for example, all analyses about the presence of an AA.

All statistical analyses were performed using IBM SPSS Statistics for Windows, version 26.0.0.1. Analyses involved group (autistic, non-autistic) as an independent variable. Binary dependent variables (‘yes’ or ‘no’) were analysed using a chi-square test of independence. Numeric outcomes (Likert-type scale data) were analysed using a Mann–Whitney *U*-test because the data were not normally distributed due to significant skewness (all Kolmogorov–Smirnov tests significant to *p* < 0.001).

### Community involvement statement

Survey questions were developed in collaboration with the members of the autistic community with lived experience of the CJS to ensure that the content of the survey was relevant. Four autistic adults took part in a focus group and provided written feedback during the design phase and final draft of the survey. The impact of autistic adults’ involvement in the design led to changes in the wording and structure of the survey to improve clarity, relevance and readability. For example, multi-part questions were made clearer by including information at the start of a question and stating the actual question directly before the space where participants add their answer. Following a focus group with autistic adults who had been involved with the CJS, we were able to include questions that were important to autistic adults’ experiences such as feeling unsupported and the mental health impact of their CJS journey.

## Results

**Figure 1.**
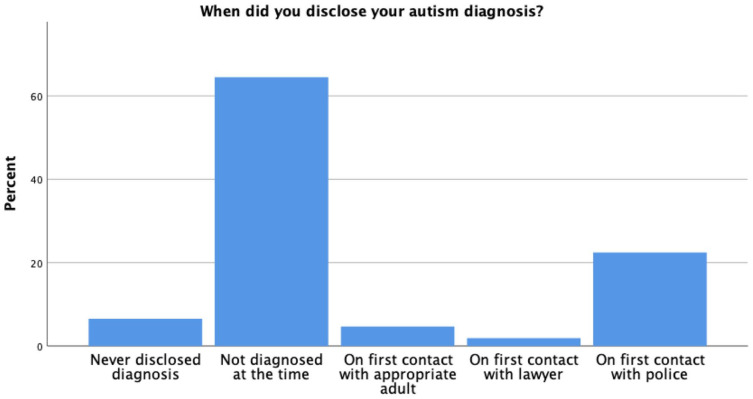
Bar graph showing the percentage breakdown regarding diagnosis disclosure based on sample of *N* = 108 participants with an autism diagnosis at the time of study completion (three missing data points).

Figure 1 shows the percentage breakdown regarding diagnosis disclosure among participants who have an autism diagnosis at the time of taking part in the study (*N* = 108). About two-thirds were not diagnosed at the time of their arrest (64%), while 7% never disclosed their autism diagnosis to anyone in the CJS, 23% disclosed their diagnosis on first contact with police, 5% on first contact with an AA and 2% on first contact with a lawyer. There may be differences in the experiences of autistic participants who did and did not have a diagnosis at the time of their involvement with the CJS. Therefore, we have run the following analyses both with (autistic *N* = 108) and without (autistic *N* = 41) autistic people who did not have a diagnosis at the time of their CJS involvement included in the autistic group.

### Autism awareness in the CJS

Autistic participants were significantly less satisfied (all *Mdn*s = 3) than non-autistic participants (all *Mdn*s = 2) with the way they were treated by police during their arrest (*U* = 6161.50, *p* = 0.004, η^2^ = 0.04), while held in custody (*U* = 6473.00, *p* = 0.03, η^2^ = 0.03) and during police questioning (*U* = 5544.00, *p* < 0.001, η^2^ = 0.07). Autistic participants (*Mdn* = 2) were also significantly less satisfied than non-autistic participants (*Mdn* = 2) with how they were treated overall by the CJS (*U* = 5638.00, *p* = 0.03, η^2^ = 0.03). Evidence for these differences was attenuated once we removed autistic participants who did not have a diagnosis prior to involvement in the CJS leaving *N* = 41 (police during arrest: *Mdn* = 2, *U* = 1800.50, *p* = 0.068, η^2^ = 0.04, held in custody: *Mdn* = 2, *U* = 1702.00, *p* = 0.024, η^2^ = 0.05, police questioning: *Mdn* = 2, *U* = 1365.50, *p* < 0.001, η^2^ = 0.10, overall: *Mdn* = 2, *U* = 1761.50, *p* = 0.376, η^2^ = 0.02.)

### Access to justice

Less than one-quarter of non-autistic participants (22/104, 21%) and similar numbers of autistic participants (28/130, 22%) stated that they had an AA present during all police interviews, χ^2^(1, *N* = 234) = 0.01, *p* = 1.0, *OR* = 1.02 (95% CI: [0.55, 1.92]). The results do not change if we only consider autistic participants who had a diagnosis at the time of arrest (12/37, 32%, χ^2^(1, *N* = 141) = 1.70, *p* = 0.33, *OR* = 1.79 (95% CI: [0.78, 4.12]). However, autistic participants (54/130, 42%) were nearly five times more likely than non-autistic participants (13/104, 13%) to state that they were not given an AA even though they believed they needed one, χ^2^(1, *N* = 234) = 23.84, *p* < 0.001, *OR* = 4.97 (95% CI: [2.53, 9.80]). These *p*-values have been corrected for the two comparisons made for these two analyses about availability of an AA. Again, the results do not change if we only consider autistic participants who had a diagnosis at the time of arrest (15/37, 41%, χ^2^(1, *N* = 141) = 13.48, *p* < 0.001, *OR* = 4.77 (95% CI: [1.99, 11.47])). The remaining participants who answered this question said that they did not have an AA but did not need one (autistic = 48/130, 37%, non-autistic = 69/104, 66%). We also asked participants who they had sought support from during their involvement in the CJS (Table 3). The most common sources of support for both autistic and non-autistic groups were from legal representatives, parents or carers, and friends.

**Table 3.** Support accessed by group.

Support type	Autism (*N* = 145)	Non-autistic (*N* = 116)
Friend	54 (37%)	53 (46%)
Parent/carer	58 (40%)	46 (40%)
Lawyer/solicitor	69 (48%)	46 (40%)
Local police service	1 (1%)	5 (4%)
Social worker	12 (8%)	5 (4%)
Healthcare provider	29 (30%)	21 (18%)
Charity organisation	14 (10%)	7 (6%)
Probation worker	16 (11%)	9 (8%)
Other	26 (18%)	11 (9%)

**Figure 2.**
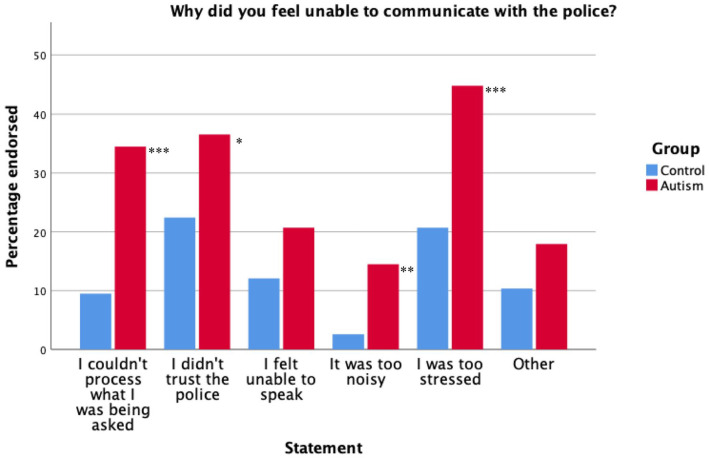
Clustered bar graph showing the percentage of endorsement for each statement. *p < 0.05, **p < 0.01, ***p < 0.001.

When asked how much they agreed with the statement ‘I felt unable to communicate with the police’, autistic participants’ agreement ratings (*Mdn* = 4) were significantly higher than non-autistics (*Mdn* = 3, *U* = 5684.50, *p* < 0.001, η^2^ = 0.07). This result did not change when only autistic participants who had a diagnosis at the time of arrest were included (*Mdn* = 4, *U* = 1554, *p* < 0.001, η^2^ = 0.08). When asked why they felt unable to communicate with the police, autistic participants were more likely to endorse the following statements: ‘I couldn’t process what I was being asked’, χ^2^(1, *N* = 261) = 22.49, *p* < 0.001, *OR* = 5.02 (95% CI: [2.47, 10.21]); *OR* = 1.99 (95% CI: [1.15, 3.46]); ‘I was too stressed’, χ^2^(1, *N* = 261) = 16.71, *p* < 0.001, *OR* = 3.12 (95% CI: [1.79, 5.43]) and ‘It was too noisy’, χ^2^(1, *N* = 261) = 10.92, *p* = 0.005, *OR* = 6.38 (95% CI: [1.85, 21.96]). No significant association was found between group and endorsement of ‘I didn’t trust the police’, χ^2^(1, *N* = 261) = 6.10, *p* = 0.07 or ‘I felt unable to speak’, χ^2^(1, *N* = 261) = 3.42, *p* = 0.32, *OR* = 1.90 (95% CI: [0.96, 3.78]). The Bonferroni correction has been applied across these five comparisons for reasons of not feeling able to communicate with the police. A clustered bar graph showing the percentage of endorsement for each statement is displayed in Figure 2.

These results did not differ when analyses were re-run only including autistic people with a diagnosis at the time of arrest, apart from: ‘I didn’t trust the police’ became significant; χ^2^(1, *N* = 157) = 11.98, *p* = 0.002, *OR* = 3.64 (95% CI: [1.71, 7.71]). ‘I couldn’t process what I was being asked’, χ^2^(1, *N* = 157) = 21.14, *p* < 0.001, *OR* = 6.76 (95% CI: [2.81, 16.28]); ‘I was too stressed’, χ^2^(1, *N* = 157) = 20.44, *p* < 0.001, *OR* = 5.41 (95% CI: [2.51, 11.65]), ‘It was too noisy’, χ^2^(1, *N* = 157) = 13.32, *p* = 0.001, *OR* = 9.13 (95% CI: [2.29, 36.38]) and ‘I felt unable to speak’, χ^2^(1, *N* = 157) = 3.55, *p* = 0.30, *OR* = 2.35 (95% CI: [0.95, 5.81]).

### Mental ill-health and CJS involvement

Both groups reported a similar frequency in relation to self-harm (non-autistic = 24%, autism = 35%) and having thoughts about ending their life (non-autistic = 42%, autism = 54%) during their CJS involvement. No significant differences were found in how much the non-autistic group (*Mdn* = 3) and the autistic group (*Mdn* = 4) agreed with the statement ‘I felt able to access the support I needed to get me through my experience’ (*U* = 4671.00, *p* = 0.08, η^2^ = 0.01). This result did not change when only autistic participants with a diagnosis at the time of arrest were included (*U* = 1961.00, *p* = 0.392, η^2^ = 0.01). This indicates that both groups disagreed with this statement and felt unable to access the support they needed. However, the autistic group (*Mdn* = 4) disagreed more strongly with the statement ‘I felt able to cope with the stress of being involved with the CJS than did the non-autistic group (*Mdn* = 4), *U* = 5475.50, *p* = 0.002, η^2^ = 0.04. This result did not change when only autistic participants with a diagnosis at the time of arrest were included (*U* = 1715.00, *p* = 0.049, η^2^ = 0.03).

### Experiences specific to autistic participants

The following analyses include only participants who have an autism diagnosis from a professional at the time of taking part in the study (i.e. not including self-diagnosed participants).

Meltdowns and shutdowns are common reactions to stress in autistic people. Meltdown is defined as an intense physical or verbal reaction to a stressful situation or environment. This may include lashing out verbally or physically at others, and may also include screaming, shouting, ranting, kicking, hitting or biting. Shutdown is a different reaction to sensory overload, stress or mental and physical exhaustion. This may include not being able to communicate in any way, going completely silent and stiff and not being able to move or respond to what is going on around you. Overall, 82% of diagnosed autistic participants reported meltdowns and 62% reported shutdowns they attributed to their CJS involvement, indicating that this is a common response in this population.

## Discussion

This study presents data from autistic and non-autistic people who have been arrested in their lifetime. A case-comparison design allowed us to examine injustices that are unique to autistic people and identify barriers that need to be addressed with policy change to make the CJS autism-inclusive. Autistic people reported significantly less satisfaction than non-autistic people regarding how they were treated overall by the CJS. Autistic participants were less satisfied with their treatment by police during arrest, while held in custody and during police questioning.

When we compared just those autistic people who had a diagnosis at the time of CJS involvement with the control group, we found less evidence for a group difference in satisfaction. This may be because having a diagnosis makes it easier for adjustments to be put in place, although we still found that this group was more likely to say they did not receive the support they required compared to controls. Alternatively, it may reflect the smaller sample size, which has reduced statistical power to find a difference between these two groups given the majority of our sample were undiagnosed at the time of their involvement. However, for all the other statistical analyses, there were no qualitative differences in the results when we removed participants who were not diagnosed at the time of arrest.

The consistency of this finding across different stages of the CJS suggests that there may be a systemic lack of autism awareness within the CJS that needs to be addressed urgently. Three areas need attention to address the inequalities faced by autistic people going through the criminal justice process: autism awareness to improve how autistic people are identified and treated, improvements to accessing justice (such as the availability of an AA) and addressing the impact on mental health. To identify issues relating to why autistic people felt less satisfied with their treatment, we asked further questions about whether reasonable adjustments were made at the police station and how the criminal justice process impacted their mental well-being.

Under the requirement for the detention, treatment and questioning of suspects in police custody outlined in PACE Code C (2019), all vulnerable adults (i.e. those with a mental disability) should be given an AA during police questioning (Home Office, 2019). It is also stated that an AA should be provided if an officer has any reason to suspect that an adult is vulnerable, which is an important instruction in cases involving individuals who may be undiagnosed at the time of their arrest or do not disclose their diagnosis. Our data indicate that less than one-quarter of our autistic participants had an AA present during all police interviews, which was similar to the proportion of non-autistic participants who had an AA present. Autistic participants who were not given an AA were also five times more likely to state that they believed they needed one, compared with the non-autistic group. From our data, we do not know the reason why participants did not receive an AA when they felt they needed one. It may be that they were not offered an AA or it may be that some chose not to have the support because they felt it could be stigmatising to ask for a support person. Furthermore, although it should be an option to have an AA who is not a family member, if this is not available, people may be concerned about the personal ramifications of involving a person close to them in their interactions with law enforcement.

One reason that may explain why we found such a high proportion of autistic participants who did not have an AA despite feeling they need one could be because the police are not adequately able to identify autistic people as they enter custody. In our sample, about two-thirds of autistic participants (diagnosed at the time of taking part in the study) were not diagnosed at the time of their arrest. In a previous study, we found that lawyers of autistic defendants reported 29% of their autistic clients received their autism diagnosis *during* criminal proceedings and 10% received their diagnosis *after* proceedings (Slavny-Cross et al., 2022). Indeed, many autistic people are undiagnosed until adulthood (Lai & Baron-Cohen, 2015) making it difficult for the police to identify them as autistic and therefore as requiring adjustments and further support. We recommend that lawyers should suggest to defendants who they suspect might have autism to seek diagnosis, as it is easier to access support once a diagnosis is in place. We also recommend that needs should be assessed irrespective of the presence of a diagnosis given the number of undiagnosed autistic adults who are likely to come into contact with the CJS. Training the police to be aware that a suspect may be autistic is also important, as they can ensure appropriate support is put in place at the earliest opportunity.

However, we acknowledge that there are challenges associated with recognising vulnerability in this undiagnosed population. First, limited resources and pressures that police services are under make this challenging. In addition, it is well documented that masking of autistic features is very common among autistic adults (Hull et al., 2017), which can make detecting neurodivergent people difficult for frontline professionals, even with relevant training.

Another barrier to identifying autistic people as they enter the CJS is that some autistic people do not disclose their autism diagnosis to the police. In our sample of autistic people, 7% never disclosed their autism diagnosis to any person in the CJS. Previous research suggests that non-disclosure of existing diagnoses may be much higher, with Crane et al. (2016) reporting that 39% of their participants did not disclose their autism diagnosis. The decision not to disclose an autism diagnosis was linked to fears that they would experience discrimination and victimisation by police officers (Crane et al. 2016). An autistic person may only disclose their autism diagnosis if they are directly asked whether they are autistic. Asking an autistic person if they have a mental disability may not illicit a disclosure of an autism diagnosis as many autistic people do not view their autism as a disability but rather, as an example of neurodiversity. Screening questions regarding diagnosed conditions should be carefully worded to avoid any ambiguity regarding the inclusion of an autism diagnosis (e.g. asking ‘have you been diagnosed with a condition that affects the way you communicate with others, such as autism?’ may avoid ambiguity).

Previous research has raised concerns regarding autistic peoples’ suggestibility and susceptibility to acquiescence during police interviews (Finlay & Lyons, 2002; Gudjonsson & Joyce, 2011; Stancliffe et al., 2015). In our sample, autistic participants were more likely than non-autistic participants to agree with the statement ‘I felt unable to communicate with the police’. When asked *why* they felt unable to communicate with the police, reasons such as finding it difficult to process what was being asked, not trusting the police, feeling ‘too stressed’ and finding the environment ‘too noisy’ were all endorsed. These findings highlight the communication difficulties that autistic people face when navigating the CJS. Previous research has cited carers of autistic people being fearful of police contact because of the potential for police to misunderstand autistic behaviours and view these interactions as non-compliance and aggressive (Wallace et al., 2021). Therefore, there may be a more systemic issue regarding autism inclusion and awareness within the CJS which needs to be addressed by involving autistic people in the training of police officers. This type of training is available in the United Kingdom, led by the National Autistic Society (see https://www.autism.org.uk/advice-and-guidance/topics/criminal-justice/criminal-justice/professionals). However, autism awareness training is not a mandatory requirement for legal professionals, so uptake of this type of training is not widespread. Another potential solution to this problem is to give training to autistic people to help them navigate interactions with police officers, for example, using virtual reality programmes (McCleery et al., 2020). Our findings also indicate the importance of providing an AA during police questioning to reduce stress triggers where possible, to reduce fear and to help an autistic person feel supported during the justice process.

Autistic people have a disproportionate risk of developing mental health difficulties such as anxiety and depression in their lifetime (Griffiths et al., 2019; Hollocks et al., 2019) and are more likely to be affected by self-harm and suicide compared to those in the general population (Hirvikoski et al., 2020; Hwang et al., 2019; Kirby et al., 2019). Our earlier study found that two-thirds of adults recently diagnosed with autism had contemplated suicide, with just more than one-third having planned or attempted suicide (Cassidy et al., 2014). Being arrested and subject to criminal proceedings is a highly stressful experience and likely has a profound impact on a person’s mental well-being. We previously found that lawyers were nearly four times more likely to be concerned that their autistic clients would engage in self-harm behaviours compared with non-autistic clients and were more likely to experience meltdowns as a result of their involvement with the CJS (Slavny-Cross et al., 2022).

The current results corroborate previous data from lawyers in that autistic participants reported experiencing significantly more meltdowns and shutdowns as a result of their CJS involvement compared with non-autistic participants. Both autistic and non-autistic participants reported similarly concerning levels of self-harm (35%, 24%) and thoughts about ending their life (54%, 42%) during the criminal justice process. However, the autistic group felt less able to cope with the stress of being involved with the CJS than the non-autistic participants did. Accessing support during involvement with the CJS is vital to prevent any impact on mental health for both autistic and non-autistic people. Both autistic and non-autistic people felt similarly unable to access the support they needed, which suggests that there is a general lack of support available. Given the high numbers of offenders reporting mental health challenges, it may be beneficial to screen for mental health and intellectual disabilities that may necessitate an AA, rather than relying on people to disclose existing diagnoses or police to recognise them.

### Limitations and conclusions

This study uses self-report data to understand the experiences of autistic people involved with the CJS and its impact on their mental health. Our inclusion criterion was any person who had been arrested at some point in their life. Therefore, we relied on participants who were reporting on events that happened several years prior to accurately recall their experiences. However, we decided to include people who had been arrested at any time in their life so as not to reduce the sampling pool of autistic people who have experience of the CJS. One limitation of the current study is that we do not have data on the date of arrest and therefore cannot know what specific legislations may have been in place at the time of arrest. The decision to not collect data on the date of arrest was made to protect participants’ anonymity, particularly for less common crimes such as terrorism offences. The majority of our participants described their ethnicity as ‘white’ which is not representative of the target population.

The majority of autistic participants in our study were undiagnosed at the time of their CJS involvement. It might be assumed that being able to disclose a clinical diagnosis would make it easier to get support. Unfortunately, we could not test whether choosing to disclose an existing diagnosis improved experience, due to the small sample size. However, we did repeat our analysis with only those with a clinical diagnosis at the time (the majority of whom choose to disclose this diagnosis) and found that autistic participants were still much more likely to feel that they did not receive the support they required compared to the control group. This suggests that having a clinical diagnosis does not remove the disadvantage faced by autistic adults in the CJS.

A strength and limitation of this study was the inclusion of participants from a number of different countries. This improved not only the generalisability of our data but also meant that participants had experience of legal systems with differing legislations and policies. To account for these differences, the survey covered broad constructs that were likely to be relevant to most jurisdictions, such as reasonable adjustments and having an independent adult present during police interviews. We included definitions of the terms used in the survey (i.e. AA) to clarify differing legal terms for the same construct to address this limitation.

Mental health difficulties are much higher among autistic people than non-autistic people (Griffiths et al., 2018; Hirvikoski et al., 2020; Hollocks et al., 2019), which is reflected in the data reported in this study. It is not possible to conclude that the difficulties faced by the autistic group are associated only with their autism as co-occurring conditions was high in this group. It is not only difficult to separate frequently co-occurring diagnoses but it is also unclear how informative this would be with respect to understanding the experiences of autistic people.

This study compliments a previous study we conducted with lawyers who had defended an autistic person between 2015 and 2020 by providing data from autistic defendants themselves to fully understand their experiences of the CJS, their ability to effectively participate in the justice process and the impact the experience had on their mental health. It is hoped that these data will be a catalyst for policy change to reduce the disadvantages faced by autistic people who encounter the CJS. Further research is needed to identify all barriers to fair justice and to generate actionable solutions to reduce the disability gap faced by autistic people.
